# Reduced Mitochondrial Adenine Nucleotide Translocase 1 (ANT1) Correlates With Aging‐Associated Airway Remodeling

**DOI:** 10.1111/acel.70264

**Published:** 2025-10-11

**Authors:** Roshani Jha, Jian Shi, Maggie J. Sedgwick, Justin Sui, Timothy E. Corcoran, Corrine R. Kliment

**Affiliations:** ^1^ Pulmonary, Allergy, Critical Care and Sleep Medicine, Department of Medicine University of Pittsburgh Pittsburgh Pennsylvania USA; ^2^ Physician Scientist Training Program University of Pittsburgh Pittsburgh Pennsylvania USA; ^3^ Cellular and Molecular Pathology Graduate Program, Department of Pathology University of Pittsburgh Pittsburgh Pennsylvania USA

**Keywords:** aging, airway, ANT1, epithelial cells

## Abstract

Aging generates a variety of phenotypes in the lungs with increased alveolar airspaces or emphysema, decreased surface area, and increased disease susceptibility. Senescence, oxidative stress, and mitochondrial dysfunction are known contributory factors. However, the underlying mechanisms promoting unhealthy aging remain unclear. Adenine nucleotide Translocase 1 (ANT1), a mitochondrial ADP/ATP transporter, is important for mitochondrial metabolism. Loss of ANT1 has been implicated in the development of pulmonary fibrosis, a disease characterized by accelerated lung aging, through mitochondrial dysfunction and senescence. To determine the role of ANT1 in normal lung aging, we analyzed aged human lung data from the Human Lung Cell Atlas and evaluated the ANT1‐related mechanism in an aged genetic mouse and in vitro models. Analysis of *SLC25A4* (ANT1) gene expression in the Human Lung Cell Atlas data from healthy adults (ages 20–80) revealed an age‐associated reduction in *SLC25A4* in alveolar type 2 pneumocytes (AT2), and airway ciliated and basal cells. Using an Ant1‐deficient mouse model, aged Ant1‐null mice developed increased airway thickening and airway resistance on lung function testing compared to aged wildtype mice. In human airway epithelial cells, ANT1 knockdown resulted in upregulation of senescence and tissue remodeling genes, including *COL8A1*. Aged Ant1‐null mice and aged human airways similarly had increased p21 expression in AT2 and airway club cells, increased SASP markers, and increased COL8A1 expression in the airways. We demonstrate for the first time that ANT1, an important multifunctional mitochondrial protein, plays a significant role in the pathogenesis of lung aging by regulating senescence and airway matrix remodeling.

## Graphical abstract

Adenine nucleotide translocase 1 (ANT1), a mitochondrial ATP transporter, is decreased in the lung epithelium of aged adults and aged mice correlating to pathogenic airway changes. Loss of ANT1 correlates with increased cellular senescence and changes in COL8A1 in the airway epithelium of the lung.

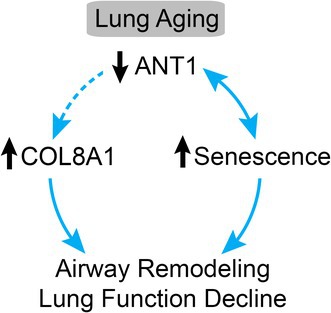

**Abbreviations**
A1KO: Ant1 knockout mice
ANT: adenine nucleotide translocase
COPD: chronic obstructive pulmonary disease
IPF: idiopathic pulmonary fibrosis
WT: wildtype

## 1 Introduction

With an ever‐increasing aging population, there are more people suffering from age‐related disorders and functional decline. Accelerated and pathogenic aging of the lung is thought to be a characteristic of chronic lung diseases such as Chronic Obstructive Pulmonary Disease (COPD) and Idiopathic Pulmonary Fibrosis (IPF). Normal aging is also marked by specific changes in the lungs, such as reduced surface area from increased alveolar spaces or emphysema, decreased lung function, and decreases in overall immune cell function (Copley et al. 2009). There is also thickening of the airways (Vikgren et al. 2004; Occhipinti et al. 2017; Weyand and Goronzy 2016) with increased airway resistance, causing reduced airflow rates. Most importantly, the cellular and molecular mechanisms that contribute to dysfunctional aging in the lung, and how we can achieve healthy lung aging, remain unknown.

Cellular senescence and mitochondrial dysfunction are hallmarks of molecular aging in numerous tissues, including the lung. Senescent cells are in a state of permanent cell cycle arrest, and they further influence neighboring cells and processes through a pro‐inflammatory senescence‐associated secretory phenotype (SASP). Overall, an accumulation of senescent cells and their SASP signals increases aging and has been demonstrated in lung diseases such as COPD and IPF (Barnes et al. 2019; Schafer et al. 2017; Sui et al. 2023). Senescence can be stimulated by factors such as DNA damage and oxidative stress. Mitochondrial dysfunction is also a driving factor of senescence and helps regulate the SASP and resist apoptosis in these affected cells. Altered mitochondrial function, including enlarged mitochondria, increased mitochondrial reactive oxygen species, and less efficient ATP production, are found in aging (Srivastava 2017).

The adenine nucleotide translocases (ANT) are highly abundant transmembrane proteins that exchange ADP and ATP across the inner mitochondrial membrane, as well as contribute to the mitochondrial permeability‐transition pore, allow ion transport, and maintain the integrity of the mitochondria (Brenner et al. 2011). ANT1 mutations, in particular, result in a number of diseases including hypertrophic cardiomyopathy, ophthalmoplegia, muscular dystrophy, bipolar disorder, and lung fibrosis (Mishra et al. 2023). In the lung, loss of ANT1 has been implicated in the development of pulmonary fibrosis, an age‐related disease characterized by progressive lung scarring, through mitochondrial dysfunction and increased senescence (Sui et al. 2023). Gaining a better understanding of the role of ANT1 in these age‐related changes will clarify how mitochondrial mechanisms influence lung aging and inform on how to promote healthy lung aging. Here, we show that aging in the lung is associated with a reduction in ANT1 expression and that this leads to increased senescence and remodeling at the lung airways.

## 2 Results

### 2.1 Expression of SLC25A4 (ANT1) Was Decreased in the Lungs of Humans and Mice With Age

**FIGURE 1:**
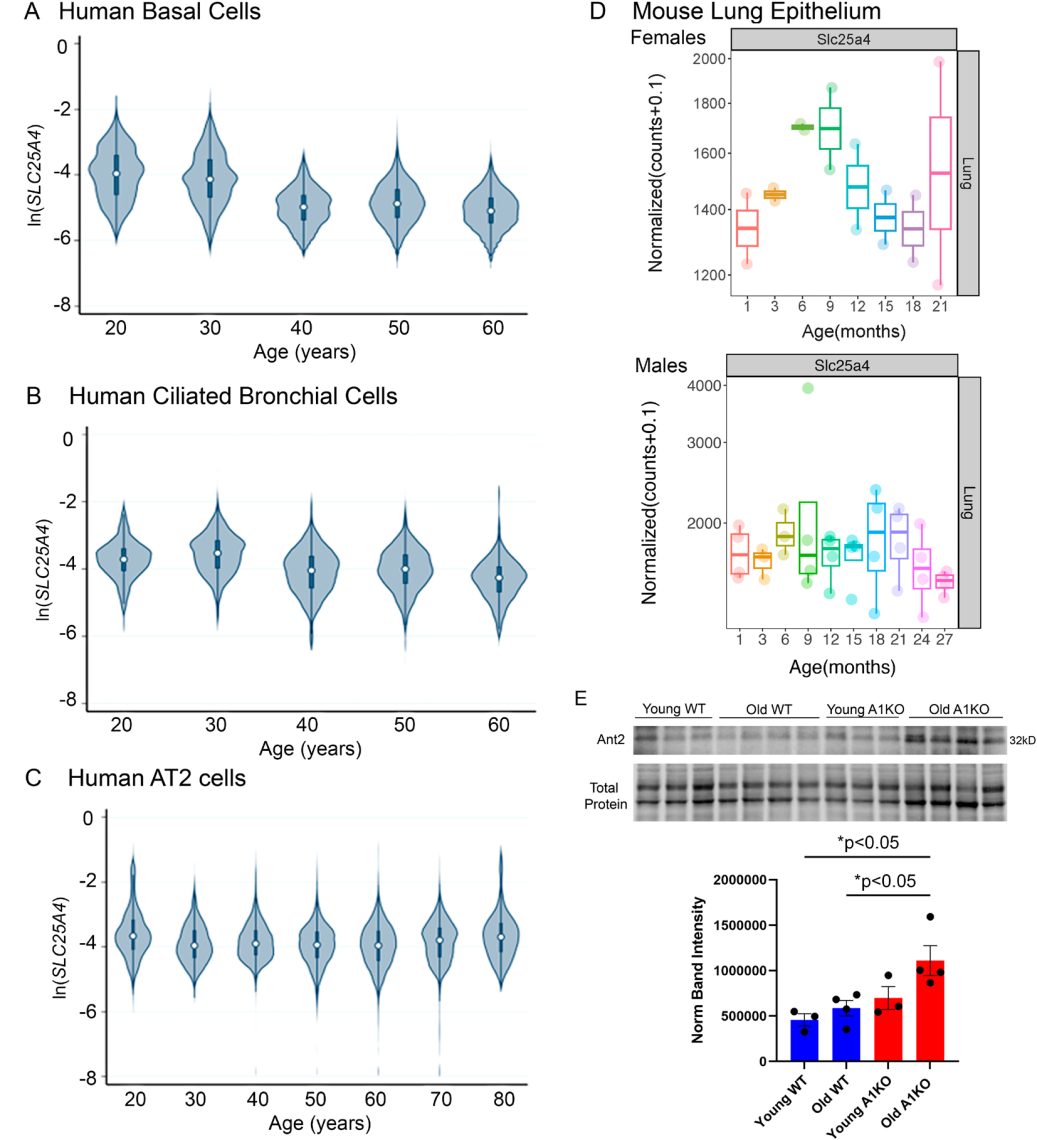
*SLC25A4* (ANT1) expression in human lungs is decreased in an age‐associated manner. (A–C) Single cell RNA sequencing from a healthy human dataset from the Human Lung Cell Atlas shows that *SLC25A4* expression (log normalized, ln) decreases with age in the (A) airway basal cells (*r* = −0.5151, *p* < 0.0001), (B) ciliated bronchial epithelial cells (*r* = −0.3246, *p* < 0.0001), and (C) AT2 cells (*r* = −0.1214, *p* < 0.0001). Tables in the Supporting Information data show the number of total cells analyzed (samples), number of human donors, number, and percentage of cells with expression of *SLC25A4* (ANT1). Statistics for panels A–C by Pearson's *r* test with *p*‐values in the legend. (D) Bulk RNA sequencing from the Tabula Muris database shows decreased *Slc25a4* expression in female and male mice lungs over age. (E) Western blot and quantification relative to total protein intensity for Ant2 from mouse lungs of wildtype and Ant1 knockout (A1KO) mice, young and old (*n* = 3–4 mice per group). *p < 0.05. Statistics by 2‐way ANOVA with Tukey's post‐test. Data are shown as mean ± SEM.

To establish whether the ANT1 expression in the lung changes with age, we looked at its gene expression (*SLC25A4*) levels in human basal cells, ciliated cells, and alveolar type II cells of human lungs with age via the single‐cell RNA sequencing from the Human Lung Cell Atlas project. We saw that *SLC25A4* expression and age were negatively correlated, especially within the basal and ciliated cells of the airways at *r* = −0.5151 and *r* = −0.3246 with *p*‐values < 0.0001 respectively (Figure 1A–C, Tables S1–S3). Tables in the Supporting Information data show the number of total cells analyzed (samples), number of human donors by sex, number and percentage of cells with expression of *SLC25A4* (ANT1), and differences in expression by sex. When analyzed by sex, *SLC25A4* expression level was increased in females in respiratory basal and ciliated cells compared to males (Table S4, basal cell median log expression: Females −9.03 vs. Males −9.36, *p < 0.0001; ciliated cell mean log expression: Females −5.66 vs. Males −5.81, *p < 0.0001; Statistics by Wilcoxon rank sum). Finally, *SLC25A4* expression level was also decreased in females in AT2 cells compared to males (Table S4, AT2 cell median log expression: Females −8.50 vs. Males −8.48, *p = 0.0082). These findings represent a significant difference within the airway cell types by sex with uncertain biological significance. We also considered the relationship between age and *SLC25A4* expression independently in male and female groups for all 3 cell types shown in Figure 1. In all cases, there were statistically significant decreases in *SLC25A4* expression with age that approximated the r values described in Figure 1. These data indicated that *SLC25A4* (ANT1) expression was reduced with age in both females and males, especially within the airway cells of the lungs.

We also looked at Ant1 expression in the context of aging in mice. Bulk RNA sequencing data from a mouse aging cell atlas database (Tabula Muris) showed decreased *Slc25a4* expression in the lungs of female mice with increased age after 9 months old and a reduction in male mice after 18 months old (Figure 1D). We found an increase in the Ant2 protein levels in the Old A1KO mice that could provide some compensation for this loss (Figure 1E). Thus, in both the human and mouse data, we show that ANT1 has a role in the aging lungs, as its levels are negatively correlated with age.

### 2.2 Loss of Ant1 Resulted in a Distinct Airway Phenotype in Aged Mice

We next sought to understand what roles ANT1 played in influencing aging and its phenotypic effects in the lungs. We analyzed global ANT1 KO mice at 2 months (young) and 22 months (old), along with their young and old wildtype (WT) counterparts. While no apparent differences in the lung were observed in young mice, we observed significant differences in the lung structure and function between the old WT and old A1KO mice.

**FIGURE 2:**
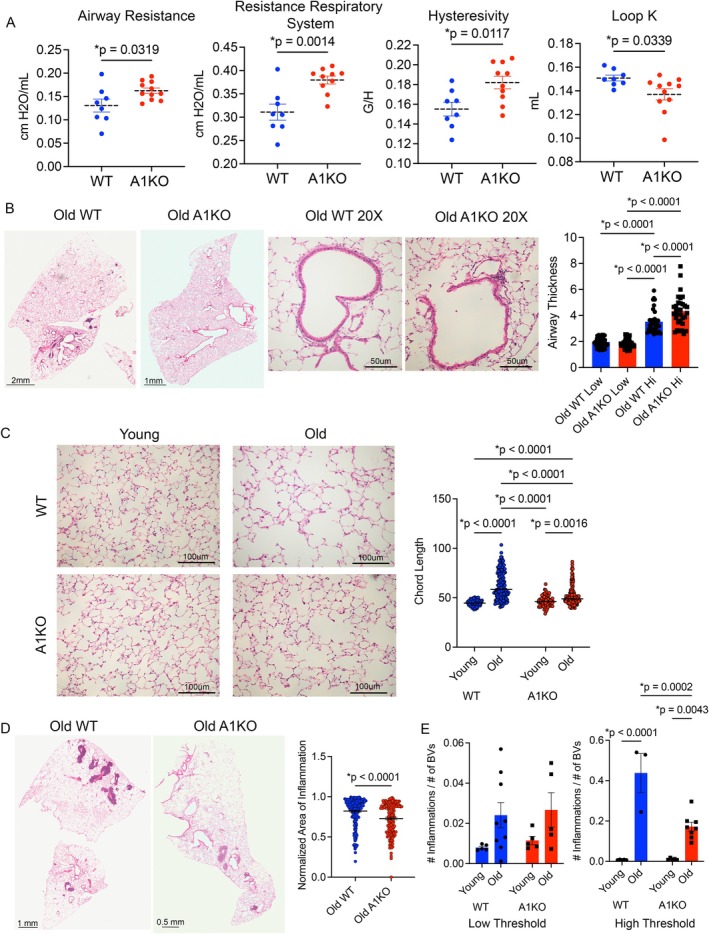
Aging of Ant1 knockout mice revealed a distinct airway phenotype. Data represent *n* = 14 for Old WT and Old A1KO mice and *n* = 5 for Young WT and Young A1KO mice. (A) Pulmonary function testing was performed on aged WT and A1KO mice (22 months). Airway resistance, resistance of the respiratory system, hysteresivity, and Loop K (volume‐independent indicator of respiratory system compliance) are shown. *p‐values are shown. (B) H&E‐stained mouse lung slices were quantified for airway thickness using Image J. After differentiating for low versus high values of thickness, the A1KO mice had more thick airways (*p* < 0.0001). Data are shown as mean ± SEM. Statistics by ANOVA with Kruskal–Wallis post‐test. (C) H&E‐stained mouse lung slices were quantified for alveolar cord length (an assessment of emphysema) shown as mean ± SEM. Statistics by 2‐way ANOVA with Tukey's post‐test. *p‐values are shown. (D) The H&E‐stained images were analyzed to determine the size of the area of inflammation surrounding blood vessels, relative to the size of the blood vessel. *p‐values are shown. Statistics were calculated using an unpaired two‐sample *t*‐test. (E) The number of inflammatory clusters on H&E staining was quantified in young and old WT and A1KO mice. *p‐values are shown. Statistics by 2‐way ANOVA with Tukey's post‐test. All data are shown as mean ± SEM.

On pulmonary function testing, aged A1KO mice demonstrated a significant increase in airway resistance and resistance of the respiratory system compared to the old WT mice (Figure 2A). This suggested that ANT1 may be altering the airway epithelium and therefore airflow in the lungs. Related to this, hysteresivity was also higher in the old A1KO cohort (Figure 2A). Hysteresivity is the ratio of tissue dampening over elastance and represents the transpulmonary pressure differences between inhalation and exhalation. Higher levels of hysteresivity in the old A1KO mice indicate increased energy loss between each inspiration and expiration cycle. Finally, Loop K values were reduced in the old A1KO mice compared to old WT mice, showing a volume‐independent lower compliance of the respiratory system that implies reduced alveolar emphysema (Figure 2A). To determine if the loss of ANT1 alters the airway structure, we analyzed airway thickness on H&E‐stained lung tissue sections. Old A1KO had a significant increase in airway thickness, regardless of the airway lumen size, compared to old WT mice (Figure 2B).

We also measured the mean alveolar chord lengths (a measurement of alveolar size). Aged WT mice developed age‐related enlargement of the alveoli as expected; however, there was a reduction in emphysema in the old A1KO mice (Figure 2C). Finally, these H&E images revealed decreased perivascular inflammation in old A1KO mice compared to old WT mice (Figure 2D). Although both groups of mice occasionally had inflammation surrounding their blood vessels, there were fewer numbers of inflammatory areas surrounding blood vessels relative to the number of blood vessels with A1KO in old mice (Figure 2E). In addition, the size of inflammation around the lung blood vessels, relative to the size of the blood vessel, was also smaller in old A1KO mice. Overall, we saw that Ant1 knockout in old mice led to morphological and functional changes in the lungs that were distinct from aged WT mice. These data indicated that loss of Ant1 contributes to airway remodeling while having protective effects against age‐related inflammation, alveolar enlargement, and destruction.

### 2.3 Aged Ant1 Knockout Mice Had Increased Markers of Senescence

**FIGURE 3:**
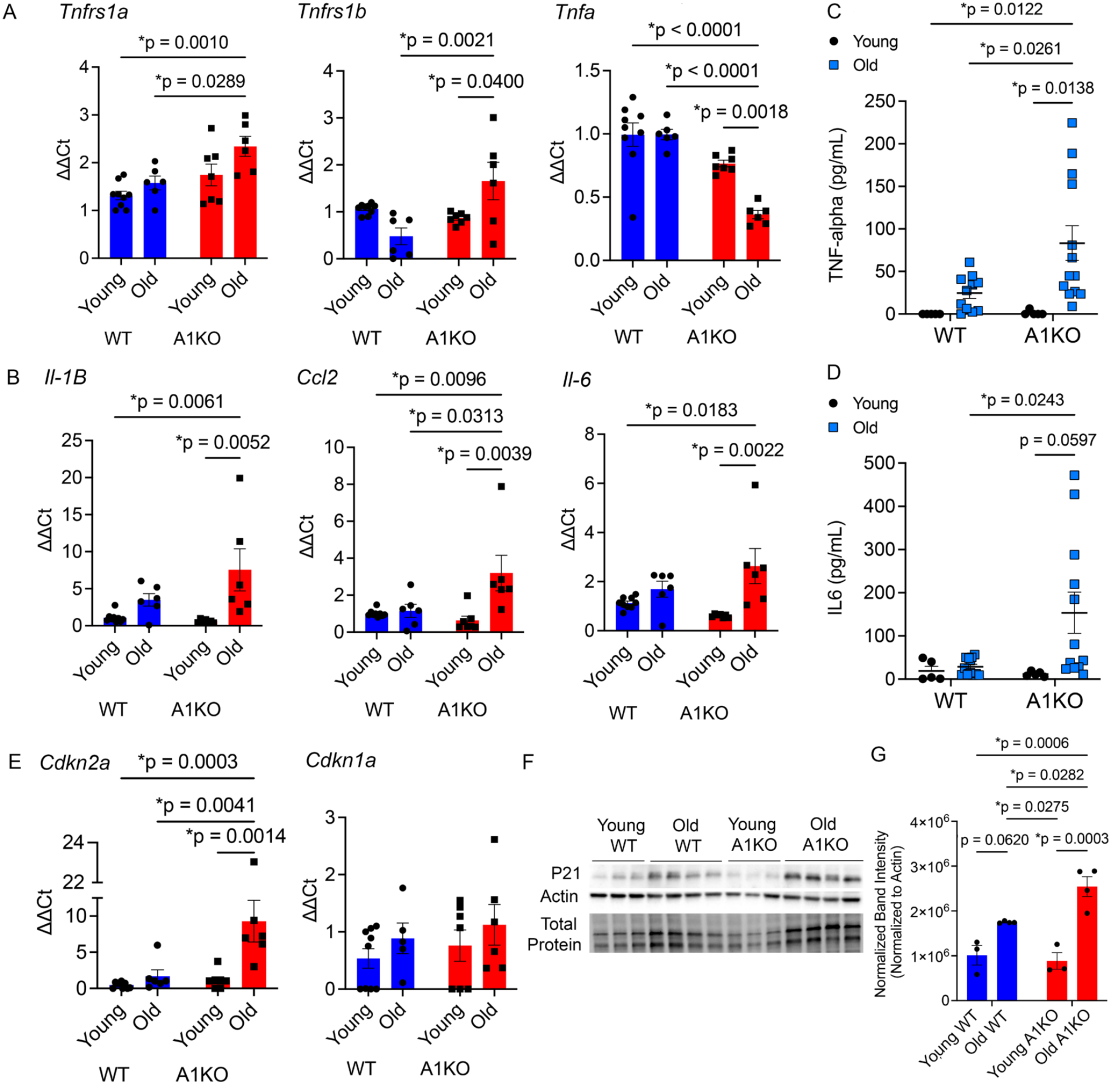
Loss of Ant1 resulted in increased senescence markers and SASP factors in aged mice. (A, B) qPCR from whole mouse lung for SASP markers. Young WT (*n* = 9), Old WT (*n* = 6), Young A1KO (*n* = 7), Old A1KO (*n* = 6). Data are shown as mean ± SEM. Statistics by Two‐way ANOVA with Tukey's post‐test. *p‐values are noted. (C, D) ELISA performed on mouse BAL for TNF‐alpha (C) and IL6 (D). Data are shown as mean ± SEM. Statistics by Two‐way ANOVA with Tukey's post‐test. *p‐values are noted. (E) qPCR from whole mouse lung for senescence markers, *Cdkn2a* (p16) and *Cdkn1a* (p21). Young WT (*n* = 9), Old WT (*n* = 6), Young A1KO (*n* = 7), Old A1KO (*n* = 6). Data are shown as mean ± SEM. Statistics by Two‐way ANOVA with Tukey's post‐test. *p‐values are noted. (F, G) Western blot with quantification from whole mouse lung analyzed for P21 in the Old A1KO mice. Results were normalized to beta‐Actin intensity per lane. Data are shown as mean ± SEM. Statistics by Two‐way ANOVA with Kruskal–Wallis post‐test. *p‐values are noted.

**FIGURE 4:**
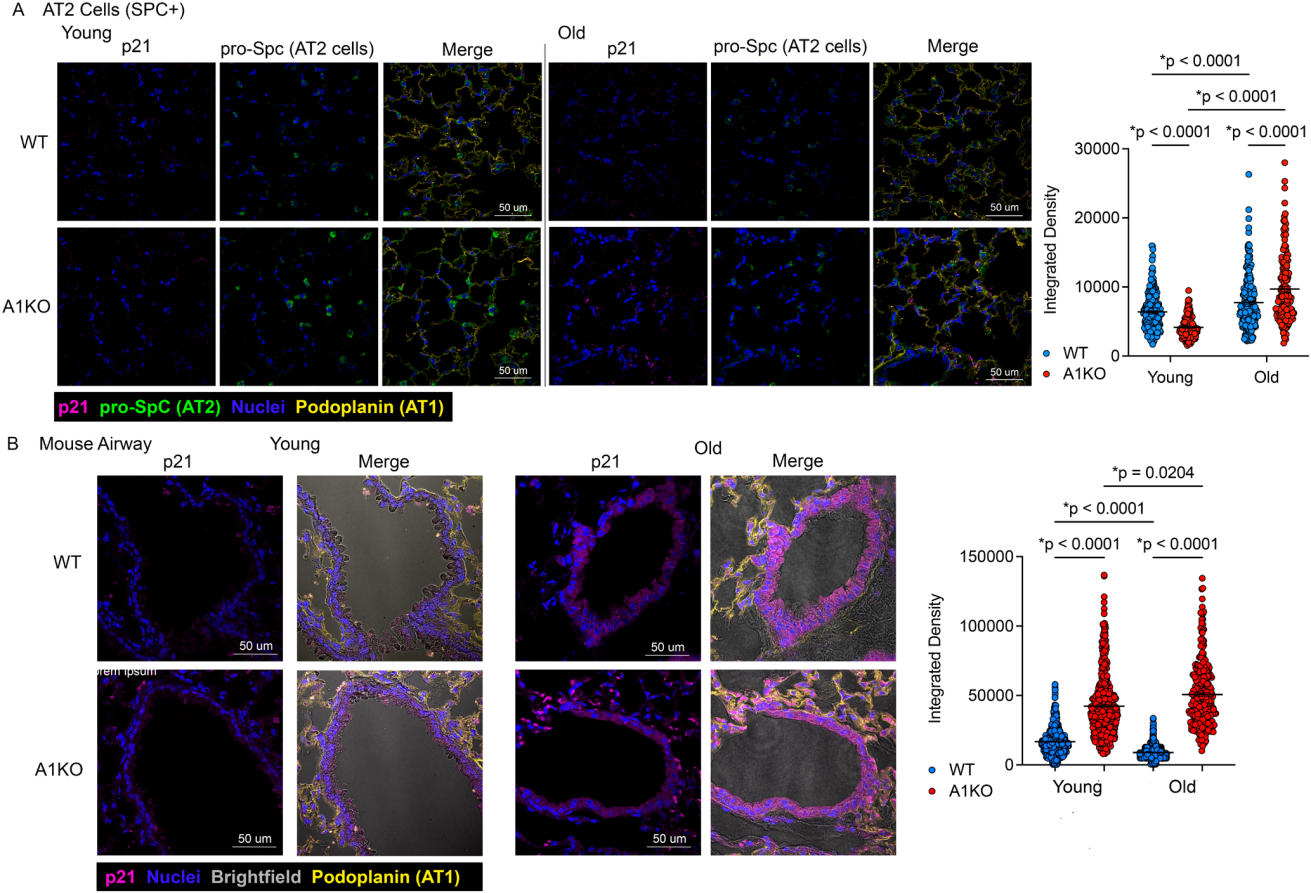
Loss of Ant1 resulted in increased senescence markers in aged mouse AT2 and airway cells. Mouse lung tissue immunofluorescence for p21 was quantified in AT2 and CC10+ airway cells (Young WT (*n* = 5), Young A1KO (*n* = 5), Old WT (*n* = 4), Old A1KO (*n* = 4)). (A) Mouse lung tissue immunofluorescence for p21 in the AT2 cells (pro‐SpC positive cells, *n* = 154–256 cells analyzed per group from 4 to 5 mice) reported as integrated density of p21 per cell. Statistics by Two‐way ANOVA with Tukey's post‐test. *p values are noted. Tissues were stained for p21, pro‐SpC, nuclei and podoplanin (AT1 marker). (B) Immunofluorescence for p21 in the CC10+ airway cells in mouse lung sections (24–35 images analyzed per group) from *n* = 4–5 mice for all groups. *p‐values are noted. Immunofluorescence data is reported as Integrated Density values for p21 from ImageJ. Statistics by Two‐way ANOVA with Tukey's post‐test. All data are shown as mean ± SEM.

We have previously shown that loss of ANT1 in lung epithelial cells resulted in increased markers of senescence and enhanced pulmonary fibrosis, a disease of accelerated lung aging (Sui et al. 2023). To determine the effect of loss of ANT1 on senescence in aging, we determined the expression of senescence markers in our aged animal lung tissue. Previous studies have also established the role of senescence and SASP markers in pulmonary fibrosis, a lung disease of aging, leading us to question the role of cellular senescence in mediating the effects of ANT1 reduction. To determine the effect of loss of ANT1 on senescence in the context of aging, we determined the expression of senescence markers in our aged animal lung tissue. We saw increased gene expression of several SASP factors including the Tnf receptor genes *Tnfrsf1a* (Tnfr2) and *Tnfrsf1b* (Tnfr2), as well as *Ccl2* in old A1KO mice compared to old WT mice by qPCR (Figure 3A,B). The gene expression of *Il‐1β* and *Il‐6* was statistically increased in old A1KO compared to young A1KO mice. We next determined if two key cytokines involved in SASP, TNF‐alpha and IL6, were altered in the bronchoalveolar lavage (BAL) of our aged mouse model at the protein level. There was a significant increase in both TNF‐alpha (Figure 3C) and IL6 (Figure 3D) in the BAL of the A1KO mice compared to young and old WT mice, supporting that there is increased production of these SASP factors. We also saw increased gene expression of the senescence marker, *Cdkn2a* (p16) in the old A1KO mice compared to young mice regardless of genotype and old WT mice (Figure 3E). While there was no statistical difference in the senescence marker Cdkn1a (p21) by gene expression, there was an increase in p21 in the whole lung tissue protein lysates of old A1KO mice compared to young WT, young A1KO, and old WT mice (Figure 3F,G). Alveolar type 2 cells (AT2) are a key progenitor cell that is important in lung repair and regeneration. p21 protein expression was also increased in AT2 cells (surfactant protein C, SPC+ cells) (Figure 4A), and in the club cells (CC10+ cells) of the airways (Figure 4B) of old A1KO mice compared to old WT mice via immunofluorescence. Overall, these results demonstrate that reduced ANT1 exerts its effects on the lungs, at least partially, through cellular senescence and its related pathways.

### 2.4 Reductions in ANT1 Led to Increased COL8A1 at the Airway Epithelium

**FIGURE 5:**
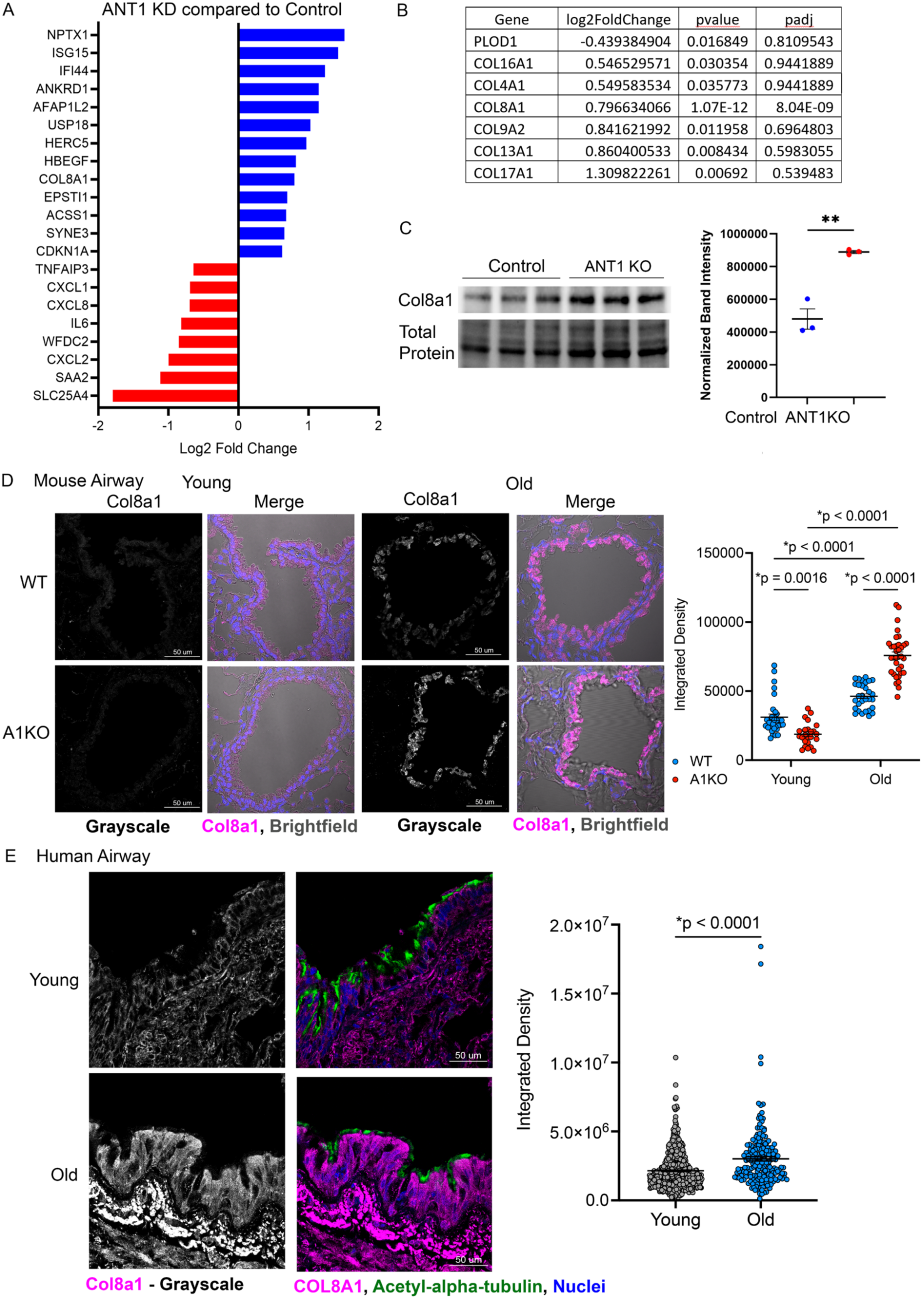
Loss of ANT1 resulted in dysregulation of collagen 8 in the airway epithelium with aging. (A) Bulk RNA sequencing was performed on human Beas‐2b cells with siRNA knockdown of ANT1 compared to non‐targeting control siRNA. Differential expression gene (DEG) analysis was performed and the top up and down‐regulated DEGs are shown with adjusted *p* < 0.05. (B) Key collagen genes that were upregulated or downregulated at a *p* < 0.05. (C) Western blot analysis of COL8A1 expression in Beas‐2b cells with Crispr‐Cas9 knockout of ANT1 compared to scrambled sgRNA control cells. Results were normalized to total protein intensity per lane. Statistics were calculated using unpaired Student's *t*‐test. **p < 0.001. (D) Immunofluorescence for Col8a1 in the CC10+ airway cells of mouse lung tissue sections. Col8a1 is shown in grayscale and composite with transmitted light brightfield, *N* = 4 for old WT and *n* = 3 for old A1KO. Data are shown as mean ± SEM. Statistics by Two‐way ANOVA with Tukey's post‐test. *p‐value noted. (E) Immunofluorescence for COL8A1 in the ciliated cells (Acetyl‐α‐tubulin+) of the airways of healthy human lungs from young (31–45 years old, *n* = 6, 2 male and 4 females) versus aged subjects (73–76 years old, *n* = 6, 3 males and 3 females) separated by age range. Immunofluorescence data is reported as Integrated Density values from ImageJ. Data are shown as mean ± SEM. *p‐value noted. Statistics were calculated using unpaired Student's *t*‐test with Mann–Whitney post‐test.

To further understand the mechanisms driving the differences in airway morphology and elevated airway resistance, we utilized a bronchial airway epithelial cell line, Beas‐2b cells with ANT1 knockdown. First, we performed bulk RNA sequencing on control Beas‐2b cells and Beas‐2b cells with ANT1 knockdown (KD) by siRNA. Among the top differentially expressed genes, *COL8A1* and *CDKN1A* (P21) were significantly upregulated in ANT1 KD cells compared to control siRNA treatment (Figure 5A). As expected, *SLC25A4* (ANT1) was the top downregulated gene with ANT1 KD. We also saw an upregulation of many collagen genes (Figure 5B). To validate our RNA seq data results, we compared cells with knockout of *SLC25A4* (ANT1) by Crispr‐cas9 and scrambled sgRNA control cells. We saw significantly increased COL8A1 protein levels (Figure 5C). We next stained for Col8a1 protein expression in the club cells of the airways of old WT and old A1KO mice. We found increased Col8a1 protein expression in the old A1KO mice compared to old WT (Figure 5D, *p < 0.0001). Finally, to confirm an age association, we evaluated human lungs from aged (ages 73–76) and young (ages 31–45) individuals without known lung disease for COL8A1 protein expression in the airway epithelium. We found that COL8A1 expression was significantly higher in the airways of the older cohort (Figure 5E, *p < 0.0001). Taken together, these results indicate that loss of ANT1 related to lung aging results in excess COL8A1 protein deposition and increased senescence in the airway epithelium and provides additional mechanism by which airway remodeling occurs with aging.

## 3 Discussion

Aging is a necessary aspect of biology; however, it is important to understand the factors that promote healthy aging and prevent functionally significant “unhealthy” or pathogenic aging. Accelerated pathogenic aging in the lungs can contribute to diseases such as COPD, pulmonary fibrosis, and lung cancer. In addition, mitochondrial dysfunction has been closely linked with aging, including changes in morphology, metabolism, and signaling. Here, we demonstrate for the first time that ANT1, a mitochondrial ATP transporter, plays a significant role in the pathogenesis of aging in the lungs. We show that ANT1 expression is decreased in the lung epithelium of aged adults and aged mice, correlating to pathogenic airway changes. Ant1‐null aged mice exhibited increased airway thickness and airway resistance on lung function testing, increased epithelial cell senescence, and increased collagen VIII accumulation in the airways. These findings support that there is an association between loss of ANT1, cellular senescence, and changes in COL8A1. We confirmed these findings in aged human airways. These findings support that a decrease in ANT1 with aging contributes to airway remodeling that may be mediated through matrix remodeling and senescence, features that contribute to age‐related lung dysfunction.

Type VIII collagen is known to be produced by endothelial cells, inflammatory cells, and corneal epithelial cells (Shuttleworth 1997). Our data support production by airway epithelial cells, suggesting that it may contribute to airway and tissue stiffness. Many studies in the lung have evaluated collagen I and IV, playing important roles in extracellular matrix remodeling in pulmonary fibrosis and chronic obstructive pulmonary disease (COPD), which are both thought to be diseases of accelerated lung aging. However, data on collagen VIII are limited. Collagen VIII was found to be elevated in serum from patients with COPD and a variety of cancers, but not pulmonary fibrosis (Hansen et al. 2016). While this study investigates collagen VIII in the context of lung epithelial cells, further study in lung endothelial cells would be very interesting and may yield important findings.

Mitochondrial dysfunction induced by loss of ANT1 also has implications in diseases of accelerated lung aging such as pulmonary fibrosis (Sui et al. 2023). Whether in aging or lung disease, it is critical to understand the cellular factors that shift healthy aging to pathogenic aging. We observed increases in senescence and COL8A1 expression with loss of ANT1, which may be further propagated by paracrine SASP factors. We hypothesize that the metabolic dysfunction and oxidative stress driven by loss of ANT1 regulate collagen expression and SASP factors such as cytokines in the airway epithelium. Future studies are necessary to understand how ANT1 expression is regulated during aging, signaling pathways connecting loss of ANT1 with matrix and cytokine changes, and if this represents a therapeutic opportunity. As little is known about the role of ANT1 in lung disease, important future directions include a clearer understanding of the effects of ANT1 by cell type, which likely differs and may result in distinct molecular signaling leading to altered cell fate decisions, SASP factor propagation, and matrix remodeling. Overexpression of ANT1 has been found to promote cellular apoptosis (Jang et al. 2008; Zamora et al. 2004; Bauer et al. 1999), suggesting that augmentation of ANT1 would need to be carefully pursued.

Previous associations of ANT1 to human health and disease mostly focus on organ systems outside of the lungs. ANT1 mutations are known to cause diseases such as cardiomyopathy, progressive external ophthalmoplegia, and bipolar disorder (Sui et al. 2023; Mishra et al. 2023). Reduced ANT1 expression has also been linked to some cancers, including rhabdomyosarcoma and breast cancer (Jang et al. 2008; Vial et al. 2020). However, our lab previously also established a link between decreased ANT1 levels and IPF (Sui et al. 2023). Overall, while ANT1 has been linked to aging‐related pathologies, particularly in IPF and cancer, we are the first to establish a link with aging in general. Airway or bronchial wall thickening has been demonstrated as an age‐related phenotype in non‐smokers (Vikgren et al. 2004). Our findings suggest that loss of ANT1 may accelerate this phenotype and may further accelerate airway disease and lung remodeling in the context of lung insults, such as cigarette smoke or air pollution, leading to lung disease. We also provide a potential mechanism for how ANT1 mediates the effects of aging in the lungs, through increased airway resistance and thickness contributed by COL8A1 and senescence. We recently published that young ANT1KO mice are protected against cigarette smoke‐induced emphysema and that this phenotype is mediated by loss of ANT1 in the monocytes and macrophages. ANT1KO monocytes and macrophages have defects in migratory capacity resulting in this protection (Sui et al. 2025). This suggests that similar defects in inflammatory cell migration occur in the aging model resulting in the altered inflammatory infiltration and reduction in air space enlargement in aging, which are consistent with the phenotype we observed in the cigarette smoke‐induced emphysema model. Overall, this research contributes to the understanding of lung aging, providing a foundation for future research into the initiation and progression of aging‐related lung diseases.

Limitations of this study include the use of global ANT1 KO mice. Since the knockout was not specific to certain cells, such as the AT2 or airway cells, we cannot determine with certainty which specific cells and tissues are driving the ANT1‐mediated changes we see. We did observe a compensatory increase in Ant2 protein expression in the lung tissue of aged Ant1‐null mice. We have previously demonstrated that increased Ant2 expression has a protective role in the lung and may therefore contribute to some of the protective phenotype aspects, such as reduced alveolar enlargement associated with aging.

In conclusion, this study provides evidence that ANT1, an abundant mitochondrial ADP/ATP transporter, plays a significant role in the pathogenesis of lung aging by regulating senescence and collagen‐mediated airway remodeling. Therapeutic targeting and augmentation of ANT1 expression may serve as a way to prevent airway remodeling during the process of lung aging.

## 4 Methods

### 4.1 Human and Mouse Genetic Studies

To determine *SLC25A4* (ANT1) gene expression changes in the lung with age, we utilized publicly available single‐cell RNA sequencing data from the cellXgene database from the Chan‐Zuckerberg Initiative (Sikkema et al. 2023). Gene expression values are reported as log normalized (ln) gene expression. Census version 2023‐12‐15 was used with the tissue type of “respiratory airway” from the “normal” disease group for the airway epithelial cell types: ciliated cells and basal cells. The “lung” tissue type was used for AT2 cells. Age data was grouped into decades from 20 to 80. Association with age was evaluated using Pearson's *r* (Megill et al. 2021). Additional analyses were performed to evaluate differences in gene expression between female and male subjects across the age spectrum. Tables in the Supporting Information data show the number of total cells analyzed (samples), number of human donors by sex, number and percentage of cells with expression of *SLC25A4* (ANT1). Using the Tabula Muris Senis (Mouse Aging Cell Atlas) database, gene expression analysis of *SLC25A4* in mouse lung tissue was performed. Bulk RNA sequencing data from mouse lung tissue was extracted from the consortium‐provided expression matrix, which reports normalized gene expression values on a log‐transformed scale. For this study, data was analyzed separately for male (ages 1–27 months) and female (ages 1–21 months) mice. Processed data and corresponding sample metadata were obtained from the publicly available portal (https://twc‐stanford.shinyapps.io/maca/).

### 4.2 Animal Studies

Animal studies were conducted with authorization from the University of Pittsburgh Institutional Animal Care and Use Committee. Ant1‐null mice (A1KO) were gifted by Douglas Wallace (University of Pennsylvania). All mice were wildtype for the *Nnt* gene. Wildtype (WT) and A1KO female mice were aged to either 2 months or 22 months, then sacrificed. Bronchoalveolar lavage was performed as previously described (Sui et al. 2023). A single right lung lobe was flash frozen for protein and RNA analysis. The remaining lung lobes were inflated with 10% formalin at 25 cm^2^ H_2_O and formalin fixed for 24 h before paraffin embedding. Serial midsagittal sections were stained with hematoxylin and eosin (H&E) for lung morphometry or for immunofluorescence. Fixed and H&E‐stained lungs were imaged at 20× magnification with brightfield microscopy. Images were captured by systematically moving through all lung fields, excluding large airways and blood vessels. 20–30 images were captured per sample for morphometric analysis. Using ImageJ software, each image was converted to gray scale and masked to exclude vascular structures and airways. Lung morphologic analysis to determine alveolar cord length (a measure of alveolar size) was completed using the script “WaffleFry” on ImageJ to create horizontal and vertical alveolar measurements, as previously described by our laboratory (Sui et al. 2023). WaffleFry can be found on Github at https://github.com/ckliment/WaffleFry.git.

### 4.3 Cell Culture and Targeted Gene Suppression

Human bronchial epithelial cell line Beas‐2b (American Type Culture Collection [ATCC]) was used. For the knockdown of *SLC25A4* (ANT1), Beas‐2b cells were transfected with 150 nM siRNA ON‐TARGETplus SMARTpools (Dharmacon: ANT1 [SLC25A4 (solute carrier family 25 member 4), catalog #L007485‐00‐0005] and control pool [catalog #D‐001810‐10‐05]) using Lipofectamine 3000 (Invitrogen). CRISPR knockout (KO) clones were generated in Beas‐2b cells using sgRNA guides (scrambled control or human *SLC25A4*) as previously described (Sui et al. 2023).

### 4.4 Human Lung Tissue

Human lung tissue (deidentified prior to receipt) acquisition and use was approved through the University of Pittsburgh Thoracic Tissue Core under IRB# 19100326‐011. Lung tissue was attained from lungs from patients who died due to accidents, without lung disease and deemed to have normal lungs as they do not exhibit any injury or any pathological abnormalities. The human lung tissue was perfused, formalin fixed, and processed for paraffin embedding. Human lung tissue samples were comprised of the following subjects (non‐smokers without known lung disease): Young (females: ages 31, 38, 41, 45 years old; males: ages 36, 42, 55 years old) and Older (females: ages 73, 73, 76 years old; males: ages 70, 73, 73 years old).

### 4.5 Tissue Immunofluorescence Staining

Immunofluorescent staining of lung tissue was completed as previously described (Sui et al. 2023). Human or mouse lung sections were sectioned at 5 μm thickness, deparaffinized, and stained for podoplanin (Developmental Studies Hybridoma Bank, Univ. of Iowa, Anti‐Pdpn no. 8.1.1), anti‐pro‐SP‐C (Millipore, cat. AB3786), COL8A1 (Invitrogen, cat. MA5‐43879), COL8A1 (Invitrogen, cat. PA5‐97604), p21 (Cell Signaling, cat. 2947), p21 (Abcam, cat. Ab107099), CC‐10 (Santa Cruz, cat. Sc‐390313), and secondary Alexa fluorophore antibodies, including goat anti‐rat Alexa 555 (Invitrogen, cat. 2089884), goat anti‐rabbit Alexa 555 (Invitrogen, cat. A32732), goat anti‐rabbit Alexa 488 (Invitrogen, cat. A32731), and goat anti‐hamster Alexa 647 (Invitrogen, cat. A21451). Control sections were stained with secondary Alexa fluorophore antibodies. All tissue sections were stained with DAPI at 10 mg/mL for 10 min. Sections were mounted with Prolong Gold (Molecular Probes, Thermo Fisher Scientific). Images were taken on a Nikon A1R confocal microscope using a 60× objective. After immunofluorescent staining, mouse lung sections were analyzed for P21 and COL8A1 expression using ImageJ (imagej.net). For P21 staining, images of alveolar parenchyma were captured by systematically moving through all lung fields, excluding large airways and blood vessels. For P21 and COL8A1 staining of the airways, images of all small to medium airways present (excluding main large airways) in the samples were captured for analysis (15–30 images per sample). For human samples, all airways present in a sample were imaged and analyzed. Using ImageJ software, each airway was captured as a region of interest (ROI), and staining intensity was normalized to ROI area.

### 4.6 Immunoblotting

Immunoblot analysis was completed on protein lysates of Beas‐2b cells and mouse lung tissue as previously described (Sui et al. 2023) using Bio‐Rad TGX stain‐free polyacrylamide gels and PVDF membranes. Primary antibodies used included ANT1 (Sigma, cat. SAB2108761), ANT2 (Cell Signaling, cat. 14671), p21 (Abcam, cat. Ab107099), COL8A1 (Invitrogen, cat. MA5‐43879), COL8A1 (Invitrogen, cat. PA5‐97604), and β‐Actin (Cell Signaling, cat. 4970T). Analysis was conducted using Image Lab Software from Bio‐Rad.

### 4.7 Bulk RNA Sequencing

Total RNA was extracted from Beas‐2b cells with ANT1 knockdown using Trizol (Thermo Fisher, Grand Island, NY) according to the manufacturer's instruction. The concentration and purity of RNA samples were measured using a NanoDrop spectrophotometer. Bulk mRNA sequencing was performed on an Illumina platform through Novogene Inc. Alignment was performed using HISAT2 (Mortazavi et al. 2008) to the reference genome. Differential expression analysis was performed using DESeq2 R package (version 1.20.0). The resulting *p*‐values were adjusted using the Benjamini and Hochberg's approach for controlling the false discovery rate. Genes with an adjusted *p* ≤ 0.05 found by DESeq2 were assigned as differentially expressed. Gene set enrichment analysis using clusterProfiler (version 3.8.1) and GSEA (version 3.0). All statistical analyses and visualizations were conducted using R (version 4.0).

### 4.8 Realtime qPCR

RNA from mouse lung tissue or Beas‐2b cells with ANT1 knockdown or knockout was extracted using Trizol and the manufacturer's instructions. RNA was converted into cDNA using the High‐Capacity cDNA Reverse Transcription Kit (Applied Biosystems, ThermoFisher) and its associated protocol. Primers used included *Cdkn1a* (Mm.PT.58.17125846), *Cdkn2a* (Mm.PT.58.8388138), *Tnfrsf1b* (Mm.PT.58.30148877), *Il‐1β* (Mm.PT.58.41616450), *Il‐6* (Mm.PT.58.10005566), *Ccl2* (Mm.PT.58.42151692), and *Gapdh* (Mm.PT.39a.1).

### 4.9 Statistics

Statistical analyses were conducted on GraphPad Prism 10 and are listed in the figure legends. Human cell expression data for Figure 1 was analyzed using Pearson's *r* test. All other data were analyzed using two‐way ANOVA and Tukey's multiple comparisons test or Student's unpaired *t*‐test with Mann–Whitney post‐test. All data is reported as mean ± SE, unless otherwise noted, and *p*‐values are shown.

## Author Contributions

Conceived the experiments: R.J., C.R.K.; Collection of samples: R.J., C.R.K.; Performed the experiments: R.J., T.E.C., M.J.S., J.S., C.R.K.; Data analysis: R.J., T.E.C., M.J.S., C.R.K.; Wrote the paper and figure creation: R.J., C.R.K.; Critical review and approval: C.R.K. and T.E.C. All authors reviewed the manuscript and approved the final version prior to submission.

## Conflicts of Interest

The authors declare no conflicts of interest.

## Supporting information


**Appendix S1:** acel70264‐sup‐0001‐AppendixS1.pdf.

## Data Availability

The data and study‐related materials will be made available upon request with the corresponding author. This article has an online data supplement, which is accessible from this issue's table of contents online. The data that support the findings of this study are available in Human Lung Cell Atlas at https://data.humancellatlas.org/hca‐bio‐networks/lung/atlases/lung‐v1‐0. These data were derived from the following resources available in the public domain:—Human Lung Cell Atlas, https://data.humancellatlas.org/hca‐bio‐networks/lung/atlases/lung‐v1‐0.
