# Supplementary material for: Reduced Mitochondrial Adenine Nucleotide Translocase 1 (ANT1) Correlates With Aging‐Associated Airway Remodeling
Source: Aging Cell. 2025 Oct 11;24(12):e70264. doi: 10.1111/acel.70264 (PMC12686547; doi:10.1111/acel.70264)
Supplement: Supplementary file 1 — Appendix S1: acel70264‐sup‐0001‐AppendixS1.pdf. [file ACEL-24-e70264-s001.pdf]

Supplemental Tables

Table 1

	SLC25A4 (ANT1) in basal cells in airway, r=-0.5151, p<0.0001							
**Decade**	**20**	**30**	**40**	**50**	**60**	**70**	**80**	**Total**
**Samples**	23,880	50,417	3,722	19,939	18,212	0	0	116,170
**Donors**	13	18	4	12	10	0	0	57<br>Male: 28<br>Female: 29
**# Expressing**	5,249	13,698	2,670	11,608	13,678	0	0	46,903
**% Expressing**	22	27	72	58	75	0	0	40

Table 2

	SLC25A4 (ANT1) in ciliated cells in airway, r=-0.3246, p<0.0001							
**Decade**	**20**	**30**	**40**	**50**	**60**	**70**	**80**	**Total**
**Samples**	8,135	20,215	1,070	14,353	3,291	0	0	47,064
**Donors**	20	26	7	24	18	0	0	95<br>Male: 49<br>Female: 46
**# Expressing**	3,392	8,526	773	8,067	2,302	0	0	23,060
**% Expressing**	42	42	72	56	70			49

Table 3

	SLC25A4 (ANT1) in AT2 cells, r=-0.1214, p<0.0001							
**Decade**	**20**	**30**	**40**	**50**	**60**	**70**	**80**	**Total**
**Samples**	18,740	5,786	27,221	32,907	25,352	8,850	1,293	120,149
**Donors**	20	5	17	18	24	11	3	98<br>Male: 52<br>Female: 46
**# Expressing**	6,190	2,371	9,438	20,600	17,177	4,084	621	60,481
**% Expressing**	33	41	35	63	68	46	48	50

Table 4

SLC25A4 (ANT1)	Basal cells<br>*p<0.0001		Ciliated cells<br>*p<0.0001		AT2 cells<br>*p=0.0082	
**Sex**	**Females**	**Males**	**Females**	**Males**	**Females**	**Males**
**Mean Log expression**	-9.03	-9.36	-5.66	-5.81	-8.50	-8.48
**Samples**	18,473	28,430	10,738	12,322	37,801	22,680
**% Expressing**	34	45	45	52	49	52
Statistics by Wilcoxon rank sum						
